# The Impact of Fluid Overload and Variation on Residual Renal Function in Peritoneal Dialysis Patient

**DOI:** 10.1371/journal.pone.0153115

**Published:** 2016-04-19

**Authors:** Na Tian, Qunying Guo, Qian Zhou, Peiyi Cao, Lingyao Hong, Menghua Chen, Xiao Yang, Xueqing Yu

**Affiliations:** 1 Department of Nephrology, The First Affiliated Hospital, Sun Yat-sen University, Key Laboratory of Nephrology, Ministry of Health and Guangdong Province, Guangzhou, China; 2 Department of Nephrology, General Hospital of Ningxia Medical University, Ningxia, China; University of Utah School of Medicine, UNITED STATES

## Abstract

**Background:**

The effect of fluid overload and variation on residual renal function (RRF) in peritoneal dialysis (PD) patients is controversial.

**Methods:**

Retrospective cohort study was designed. One-hundred and ninety PD patients with measured glomerular filtration rate (mGFR) ≧ 3ml/min/1.73m^2^ were recruit. Fluid status of every participant was assessed by bioelectrical impedance analysis (BIA) every 3 months for 1 year. The cohort was divided into three hydration groups, namely persistent overhydration (PO) group, intermittent overhydration (IO) group and normal hydration (NH) group. Additionally, participants were also divided into high or low fluid variation groups. The decline rate of RRF and the event of anuria were followed up for 1 year. The association of fluid overload with RRF loss was evaluated by Cox proportional hazard models adjusted for confounders.

**Results:**

Thirty-six (18.9%) patients developed anuria. The decline rate of mGFR in both PO and IO groups were significantly faster than that of NH group (PO vs NH: -0.2 vs -0.1 ml/min/1.73m^2^/month, p < 0.01; IO vs NH: -0.2 vs -0.1 ml/min/1.73m^2^/month, p < 0.01). Kaplan-Meier analysis showed poorer RRF outcome in both PO and IO groups compared with that of NH group (PO vs NH: p < 0.001; IO vs NH: p = 0.006). Patients with high fluid variation had worse RRF survival than those with low fluid variation (p = 0.04). Adjusted Cox regression models indicated the hazard ratio of RRF loss in PO group was 8.90-folds higher (95% confidence interval 3.07–31.89) than that in NH group.

**Conclusions:**

These findings suggested fluid overload was independently associated with the decline of RRF in PD patients.

## Introduction

Residual renal function (RRF) is well recognized as a crucial factor for mortality [1], protein–energy wasting [2,3],anemia [4], inflammation [5], and technique failure [6] in patients undergoing peritoneal dialysis (PD). Therefore, preservation of RRF is still the primary goal of high quality of PD management, even in those patients undergoing long-term PD with less RRF.

Many risk factors were reported to be associated with RRF loss, including hemodialysis (HD) vs PD modality [7], mean arterial pressure [8], baseline of RRF [9], oxidative stress [10] and nephrotoxic drugs [11], and etc. However, the effect of fluid status on RRF is controversial. Intravascular volume depletion and hypotension were reported to cause a loss of RRF [7,12]. Strict volume control with salt and water restriction was found to lead to dramatic reduction in urine output [13]. Based on these findings, some clinicians even believe “slight hypervolemia” should be maintained to protect RRF [13,14]. However, Rodriguez-Carmona group [15] reported that fluid overload resulting from less ultrafiltration and sodium removal was related to faster RRF decline. Furthermore, McCafferty group [16] recently found that expanded volume was not associated with preservation of RRF.

Our previous cross-sectional study showed that there was an inverse association between residual urine output and fluid overload [8], but the effect of fluid overload on the decline of RRF in PD patients was still unclear. The purpose of this retrospective cohort study is to explore the influence of fluid overload and its variation on the decline of RRF in patients undergoing PD therapy.

## Materials and Methods

### Study Objectives

A retrospective cohort study was designed to identify associations between fluid status (both fluid overload and fluid variation) and the decline of RRF in patients undergoing PD therapy in order to find out which conditions should alert the clinician to potential RRF decline.

### Participants

Prevalent patients at PD center in The First Affiliated Hospital of Sun Yat-sen University in Guangzhou, China from November 1^st^, 2007 to March 30^th^, 2014, were assessed for eligibility for inclusion. The patient inclusion criteria were: (1) undergoing stable PD therapy ≧ 3 months; (2) age ≧ 18 years; (3) measured glomerular filtration rate (mGFR) ≧ 3ml/min/1.73m^2^ [12,17]; (4) bioelectrical impedance analysis (BIA) every 3 months for 1 year were completed; (5) signed informed consent form. The exclusion criteria were: (1) patients with pacemakers, amputation, or not able to accomplish the analysis of body composition in standing position for 3 minutes; (2) patients who had been prescribed nephrotoxic medication before or during the study period for any reasons; (3) patients with severe heart failure (New York Heart Association Class IV); (4) patients who had peritonitis within one month prior of the study enrollment. The study protocol had been approved by the Ethics Committee of The First Affiliated Hospital of Sun Yat-sen University.

### Assessment of Fluid Status and Patients Grouping

Fluid status was measured by multi-frequency bioelectrical impedance analysis (BIA) device—InBody 720 (Biospace, Seoul, Korea). InBody 720 used state-of-the-art technology and 8-point tactile electrode system to measure the total and segmental impedance and phase angle of alternating electric current at six different frequencies (1kHz, 5kHz, 50kHz, 250kHz, 500kHz, and 1000kHz). All the subjects were performed with BIA in the morning during a routine clinical visit as previously described [18]. Peritoneal dialysis fluid was not drained from the abdomen [19,20]. When the measurement was carried on, the subject stood in an upright position, on four-foot electrodes on the platform, and gripped two Palm-and-Thumb electrodes according to the manufacturer’s instruction. The results of this tool closely correlate with the gold standard measurement by isotope dilution [21,22,23]. A total of 5 measurements (every 3 months for 1 year) were recorded for each patient.

The ratio of extracellular water (ECW) / total body water (TBW) ≧ 0.4 (suggested by the manufacturer, Biospace, Seoul, Korea) was defined as fluid overload. This cut-off was set as previously described [8]. Patients with ECW/TBW<0.4 in all the five measurements were defined as normal hydration (NH) group, while the patients with at least one measurement of ECW/TBW ≧ 0.40 were defined as overhydration patients. The overhydrated patients were further divided into persistent overhydration (PO) group (defined as ECW/TBW ≧ 0.40 in all the 5 measurements with BIA) and intermittent overhydration (IO) group (defined as at least one measurement of ECW/TBW ≧ 0.4 but not all the 5 measurements).

Variation of fluid status for each patient was presented by standard deviation (SD) of ECW/TBW, which was calculated by the five repeatedly measured ECW/TBW values at the five time points (baseline, 3^rd^ month, 6^th^ month, 9^th^ month and 12^th^ month). The cut point of fluid variation was determined by restricted cubic spline method and Kaplan-Meier method (event as anuria), which was found to be 0.0044. The participants with SD of ECW/TBW ≧ 0.0044 were defined as high fluid variation group, and those with SD of ECW/TBW <0.0044 were defined as low fluid variation group.

### Data Collection

All participants’ clinical manifestations, fluid status, medication and PD prescription (PD modality, PD dosage and dialysate glucose concentration) were evaluated during their routine clinical visits. Blood was taken from each participant after an overnight fasting to measure biochemical parameters, including hemoglobin, high sensitive C reaction protein (hs-CRP), sodium, fasting glucose, urea, creatinine, albumin, phosphate, calcium, and parathyroid hormone (PTH). All the serum and dialysate biochemical parameters were measured by BECKMAN CONLTER AU5821-2 automatic biomedical analyzer (BECKMAN, USA).

All the PD patients in our center were educated to precisely measure and record the urine volume and PD solution filled in and drained out at each exchange and then calculate the 24-hour urine volume and utrafiltration everyday. In this study, 24-hour urine volume and peritoneal fluid ultrafiltration were calculated from the patient’s records one week prior to the clinic visit as a daily mean of 24-hour urine and ultrafiltration. Both 24-hour urine and PD effluent were collected at the same day to calculate Kt/V, normalized protein clearance rate (nPCR) and mGFR using Adequest 2.0 software (Baxter Healthcare, USA). The average daily glucose concentration exposure (%) was calculated by the total amount of glucose (grams) of PD solution divided by the total amount of solution (liters) used over a day. The calculation was done by averaging the past week of glucose exposure prior to the BIA measurements.

Hypotension event (defined as either SBP or DBP decreased below 90/60mmHg or 30mmHg lower than the baseline, accompanying with symptoms as dizziness, fatigue, syncope, and etc.) was collected by the clinical visit and telephone follow-up. Peritonitis episodes diagnosed as the previous described diagnostic criteria [24].

All the patients were undergoing continuous ambulatory peritoneal dialysis (CAPD) with dialysis systems from Baxter Healthcare Corp. No patient performed automated peritoneal dialysis (APD). The CAPD regimen was prescribed with 2 liters per exchange and three to five exchanges every day according to the PD adequacy of each patient. Icodextrin was not used in this program because it is not available in China yet.

### RRF Calculation

mGFR was calculated as the average clearance of 24-hour renal creatinine and urea, and normalized to 1.73m^2^ body surface area using the software of Adequest 2.0 (Baxter Healthcare, USA). Both mGFR and urine output were measured at the beginning, then every 3 months for 12 months to follow up the RRF decline rate and onset of anuria (urine volume ≦100ml/24h). RRF loss was defined as anuria.

### Management of Fluid Overload

Patients with increased ECW/TBW ratio while without decreased intracellular water (ICW) were asked to restrict salt intake (< 5 g/day) [25] and water intake. Doctors would prescribe diuretics to enhance their urine output and optimize dialysis prescription to increase their sodium and water removal, including by using more hypertonic dialysate, shortening night dwelling time, or undergoing intermittent PD.

### Statistical Analysis

The patient’s characteristics were presented as mean ± SD normally distributed continuous variables, median (interquartile range) for skewed continuous variables, frequencies and percentages for categorical variables. An independent sample *t*-test was performed for a comparison of normally distributed continuous variables. While a comparison of non-normally distributed continuous variables was performed using Mann-Whitney *U*-test. For categorical variables, Chi-square test was used. The decline rate of mGFR at all time point was calculated by simple linear regression analysis for each patient. RRF survival was calculated using the Kaplan-Meier method, and differences between distributions of survival of different groups were assessed by Breslow and Log-rank test. Meanwhile the significance level was adjusted to 0.05/3 = 0.017 in the multiple comparisons on Log-Rank test by Bonferroni method. The COX proportional hazard analysis was used to evaluate the risk factors associated with the loss of RRF, and factors with *p* value<0.1 in univariate analysis were entered into the multivariable analysis. Mean ECW/TBW was included as a continuous variable of candidate risk factor for RRF loss. A sequential series of COX proportional hazard models (model 1 through 3) were constructed to explore the relationship between PO and IO fluid status with loss of RRF. Potential confounders were considered, including those demonstrated imbalance at baseline between groups with different fluid status and for importance of clinical concerns. In order to detect multicollinearity among variables in those multivariable Cox models, collinearity diagnosis using variance inflation factor (VIF) was conducted. VIF > 10 indicates that severe multicollinearity effects are present. In the current study, all VIFs were less than 2, which meant covariate was not likely to be multicollinear to one or more of the other variables. A two-sided *p* value < 0.05 was considered statistically significant. Statistical analyses were performed using SPSS 16.0 software (SPSS, Inc., Chicago, IL), SAS 9.2 (SAS Institute Inc., Cary, USA) and R software (3.0.1).

## Results

### Patients Enrolled in the Study

**Fig 1:**
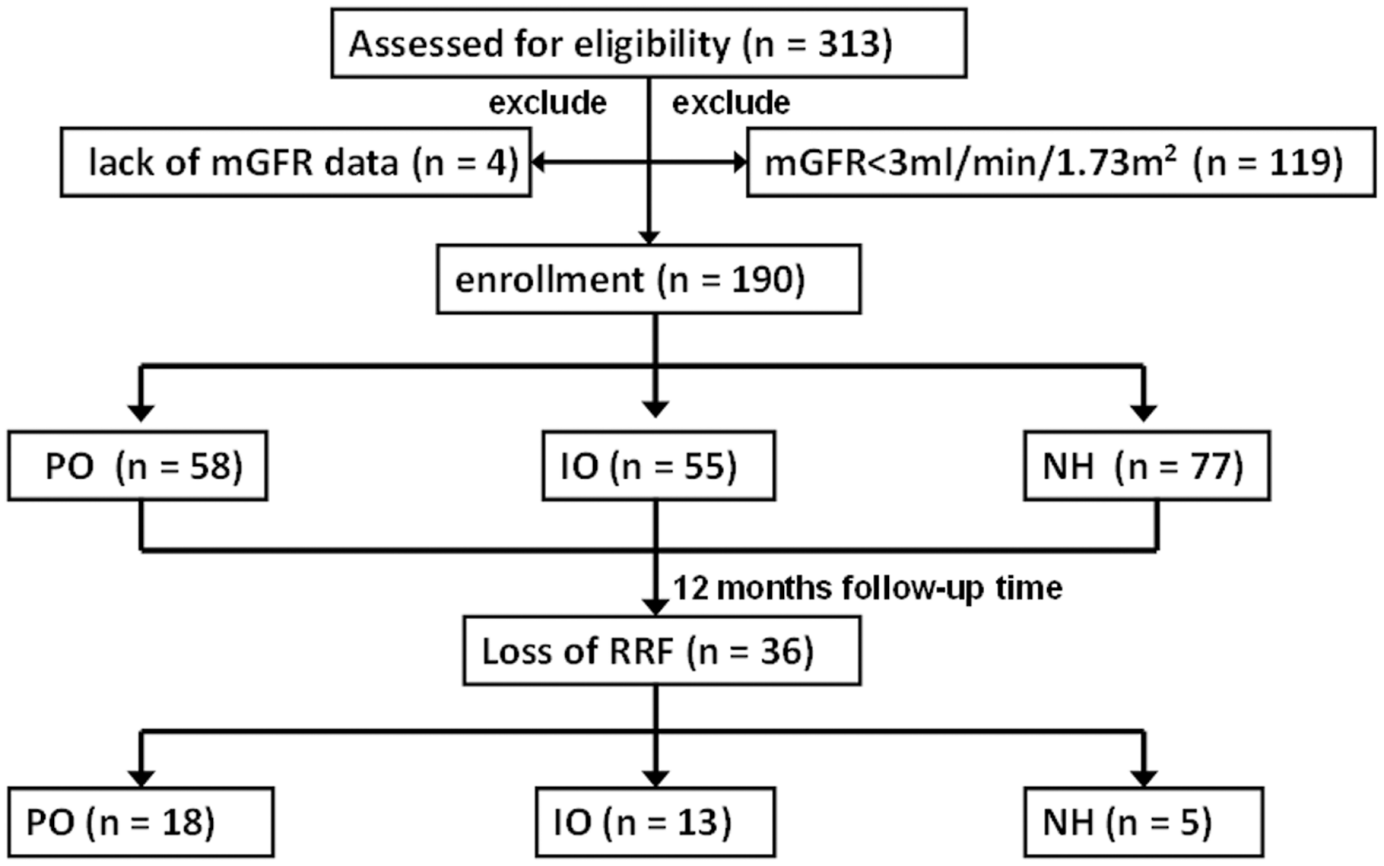
Patient enrollment and follow-up flow diagram.

Regular BIA measurements were performed in 313 PD patients for more than one year. One hundred twenty-three were excluded from this study, including four patient’s lack of mGFR data, and 119 patients with mGFR less than 3ml/min/1.73m^2^. Finally, a total of 190 prevalent patients with PD vintage of 7 (3, 20) months were enrolled in this study (Fig 1). The mean age was 47.7 ± 15.4 years old and 58% of participants were male. The underlying kidney diseases were chronic glomerulonephritis (98, 52%), diabetic nephropathy (46, 24%), hypertensive nephrosclerosis (13, 7%) and other miscellaneous causes (33, 17%). The patients were grouped according to their fluid status, including 77 (41%) patients in NH group, 58 (30%) in PO group, and 55 (29%) in IO group respectively. Among the fluid variation groups, 88 (88/190, 46%) patients were with high fluid variation and 102 (102/190, 54%) with low fluid variation. No patient was transferred from PD to HD or received transplantation during the study.

### Baseline Characteristics

Patients in PO group were older, higher percentage of diabetes and faster peritoneal transporter, more co-morbidities, lower level of hemoglobin and serum albumin. While the baseline of mGFR and urine volume were not significantly different among the three groups (data shown in Table 1).

**Table 1:** Baseline characteristics of different fluid status groups.

	Total	NH	Fluid overload		*p*
			PO	IO	
n	190	77	58	55	
Male n, (%)	111(58)	45(58)	34(59)	32(58)	0.9
Age (year) ^a^^,^^c^^,^^f^	47.7±15.4	42.2±12.4	55.7±15.5	46.9±15.7	<0.001
Vintage (month)	7(3,20)	7(3,16)	9(3,25)	6(3,20)	0.7
Diabetes n, (%) ^a^^,^^c^^,^^e^	54(28)	9(11)	35(60)	10(18)	<0.001
Body mass index (kg/m^2^)	22.0±3.3	21.6±3.1	22.4±3.9	22.2±3.1	0.3
SBP (mmHg) ^a^^,^^b^^,^^d^	136.7±20.7	129.3±17.1	142.4±21.5	141.2±21.7	<0.001
DBP (mmHg)	83.9±12.8	83.9±11.3	81.5±13.6	86.5±13.4	0.1
Charlson co-morbidity score ^a^^,^^c^^,^^d^	3(2,5)	2(2,3)	5(4,6)	3(2,5)	<0.001
mGFR (ml/min/per1.73m^2^)	3.9(3.5,5.6)	4.4(3.5,6.5)	3.9(3.5,5.4)	3.9(3.4,4.6)	0.1
Urine volume (ml/24h)	800(500,1262)	1000(575,1300)	775(400,1200)	800(550,1200)	0.2
Ultrafiltration (ml/24h)	300(0,600)	200(0,575)	400(0,662)	250(0,600)	0.5
Total output (ml/24h)	1250(925,1600)	1350(950,1650)	1200(937,1585)	1250(850,1500)	0.5
Total Kt/v	2.5±0.7	2.6±0.6	2.5±0.9	2.4±0.7	0.6
Transport type n, (%) ^a^^,^^e^ (n = 171)					0.05
H+HA	108(63)	40(55)	38(76)	30(63)	
L+LA	63(37)	33(45)	12(24)	18(37)	
Mean ECW (L)	14.6±2.4	13.9±2.6	15.6±3.0	14.6±2.5	0.002
Mean ICW (L)	21.9±3.9	21.5±4.1	22.4±3.8	21.9±3.8	0.3
Skeleton muscle mass (kg)	27.6±6.3	27.4±6.1	28.3±5.4	27.0±6.3	0.5
Body fat (kg)	12.8±7.1	12.6±7.3	13.3±7.7	12.5±6.3	0.8
Mean ECW/TBW ^a^^,^^b^^,^^d^	0.398 (0.390,0.406)	0.389 (0.386,0.392)	0.410 (0.407,0.415)	0.399 (0.397,0.402)	<0.001
SD of ECW/TBW ^a^^,^^b^^,^^c^^,^^d^	0.0039 (0.0025,0.0061)	0.0034 (0.0021,0.0049)	0.0039 (0.0025,0.0064)	0.0049 (0.0033,0.0081)	<0.001
Hemoglobin (g/L) ^a^^,^^b^^,^^f^	111.6±19.5	119.8±16.9	104.2±17.5	108.3±20.9	<0.001
hs-CRP (mg/L)	1.7(0.7,6.3)	1.4(0.7,4.7)	2.9(0.7,8.9)	1.9(0.7,4.6)	0.2
Serum sodium (mmol/L)	139.9±3.8	139.9±3.8	139.8±3.8	140.0±3.8	0.9
Fasting serum glucose (mmol/L) ^a^^,^^c^^,^^f^	5.9±2.7	5.4±2.6	6.8±3.1	5.6±2.1	0.005
Serum albumin(g/L) ^a^^,^^b^^,^^c^^,^^f^	39.2±4.7	41.2±2.9	36.8±5.8	39.0±4.2	<0.001
Hypoalbuminemia n, (%) ^a^^,^^b^^,^^c^^,^^e^	21(11)	1(1)	14(24)	6(11)	<0.001
nPCR ^a^^,^^b^^,^^f^	0.9±0.2	1.0±0.3	0.9±0.2	0.9±0.2	0.02
Serum phosphorus (mmol/L)	1.5±0.4	1.4±0.4	1.4±0.4	1.5±0.4	0.3
Serum calcium (mmol/L)	2.4±0.6	2.5±0.9	2.4±0.2	2.3±0.2	0.1
Serum PTH (pg/ml)	298(120,497)	302(126,427)	232(59,466)	358(159,527)	0.2
Sodium restriction n, (%) ^a^^,^^b^^,^^c^^,^^e^	105(55)	17(22)	53(91)	35(63)	<0.001
PD dosage (6L/8L/10L, %)	24/75/1	32/68/0	12/86/2	25/73/2	0.1
PD solution glucose concentration (%) ^a^^,^^b^^,^^f^	1.7±0.3	1.6±0.2	1.7±0.3	1.7±0.3	0.001
Antihypertensive drug category n, (%) ^a^^,^^b^^,^^c^^,^^e^					
0~2	109(57)	56(73)	22(38)	31(56)	<0.001
≧3	82(43)	21(27)	36(62)	31(44)	
Diuretics use n, (%)	17(8.9)	7(9.1)	6(10.3)	4(7.3)	0.8
ACEI/ ARB use n, (%)	104(55)	36(47)	38(66)	30(55)	0.1

Note: *p* < 0.05 for

^a^ PO versus NH,

^b^ IO versus NH;

^c^ PO versus IO.

Analyzed by the

^d^ non-parametric test,

^e^ chi-square test,

^f^ one-way ANOVA.

Abbreviations: PO: persistent overhydration, IO: intermittent overhydration, NH: normal hydration group, SBP:systolic blood pressure, DBP: diastolic blood pressure, mGFR: measured glomerular filtration rate, H: high transport type, HA: high average transport type, L: low transport type, LA: low average transport type, ECW: extracellular water, TBW: total body water, ICW: intracellular water, SD: standard deviation, hs-CRP: high sensitive C reaction protein, nPCR: normalized protein clearance rate, PTH: parathyroid hormone, ACEI:angiotensin-converting enzyme inhibitor, ARB: angiotensin receptor blocker.

Totally, 12 episodes of peritonitis were occurred in this study and no significant difference was shown among the three groups (NH vs PO vs IO: 6 (9.5%) vs 2 (4.2%) vs 4 (9.8%), *p* = 0.51). A total of 12 events of hypotension were recorded, with 5 (7.9%), 4 (8.3%) and 3 (7.3%) in NH, PO and IO group, respectively, and also there was no significant statistical difference among the three groups (*p* = 0.98).

### Changes of RRF

At the end of follow-up, patients in both PO and IO groups had worse mGFR (PO vs NH: 1.6 vs 3.1 ml/min/1.73m^2^, *p* < 0.001; IO vs NH: 1.6 vs 3.1 ml/min/1.73m^2^, *p* < 0.001) and less urine output (PO vs NH: 350 vs 800 ml/24h, *p* < 0.001; IO vs NH: 500 vs 800 ml/24h, *p* < 0.001) than those of NH group. Both the decline rate of mGFR and urine volume in the PO and IO groups were significantly faster than those in NH group (median of mGFR decline rate: PO vs NH: -0.2 vs -0.1 ml/min/1.73m^2^/month, *p* < 0.01, IO vs NH: -0.2 vs -0.1 ml/min/1.73m^2^/month, *p* < 0.01; median of urine volume decline rate: PO vs NH: -30.0 vs -16.7 ml/24h/month, *p* < 0.01; IO vs NH: -27.5 vs -16.7 ml/24h/month, *p* < 0.01) (Table 2).

**Table 2:** Change of mGFR and urine volume of different fluid status groups.

	NH (N = 77)	PO (N = 58)	IO (N = 55)	*P* value
*mGFR (ml/min/1*.*73m*^*2*^*)*				
Baseline	4.4(3.5,6.5)	3.9(3.5,5.4)	3.9(3.4,4.6)	0.1
1 year follow-up ^a^^,^^b^	3.1(1.8, 4.4)	1.6(0.4, 3.3)	1.6(0.3, 3.4)	<0.001
mGFR decline rate (per month) ^c^^,^^d^	-0.1(-0.3, 0)	-0.2(-0.3, -0.2)	-0.2(-0.3, -0.1)	0.006
*Urine volume (ml/24h)*				
Baseline	1000(575,1300)	775(400,1200)	800(550,1200)	0.2
1 year follow-up ^a^^,^^b^	800(500, 1200)	350(87, 800)	500(100, 800)	<0.001
Urine volume decline rate (per month) ^c^^,^^d^	-16.7(-33.3, 5.0)	-30.0(-53.5, -8.3)	-27.5(-50.0, -4.2)	0.007

Note: *p*<0.001

^a^ PO vs NH,

^b^ IO vs NH; *p*<0.01

^c^ PO vs NH,

^d^ IO vs NH. Analyzed by non-parametric test.

The rate of decline of mGFR and urine volume was calculated by simple linear regression analysis for each patient.

Abbreviations: PO: persistent overhydration, IO: intermittent overhydration, NH: normal hydration group, mGFR: measured glomerular filtration rate.

### Fluid Status and RRF Survival

**Fig 2:**
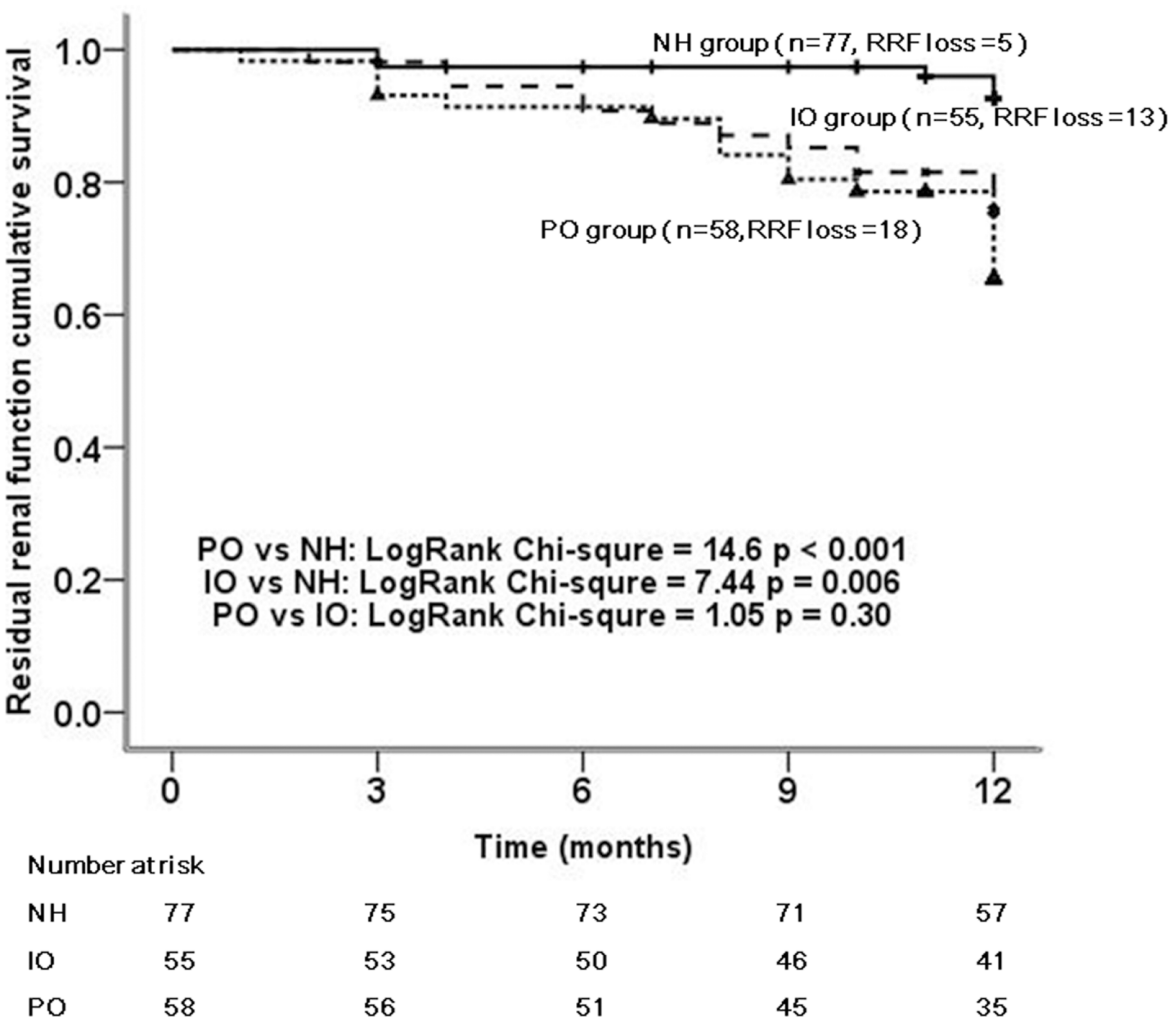
Kaplan-Meier survival curves for the residual renal function survival (event defined as anuria) in the persistent overhydration (PO) group, intermittent overhydration (IO) group, and the normal hydration (NH) group. In the multiple comparisons, the level of test was adjusted to 0.05/3 = 0.017.

Totally, 36 (19%) patients developed anuria, 18 (31%) cases in the PO group, 13 (24%) in the IO group, and 5 (6%) in the NH group, respectively (Fig 1). The Kaplan-Meier curve for RRF loss showed that the patients in PO and IO groups had poorer RRF survival rate compared with that of NH patients (PO vs NH: Log-rank chi-square = 14.6, *p* < 0.001; IO vs NH: Log-rank chi-square = 7.4, *p* = 0.006) (Fig 2).

### Fluid Variation and RRF Survival

**Fig 3:**
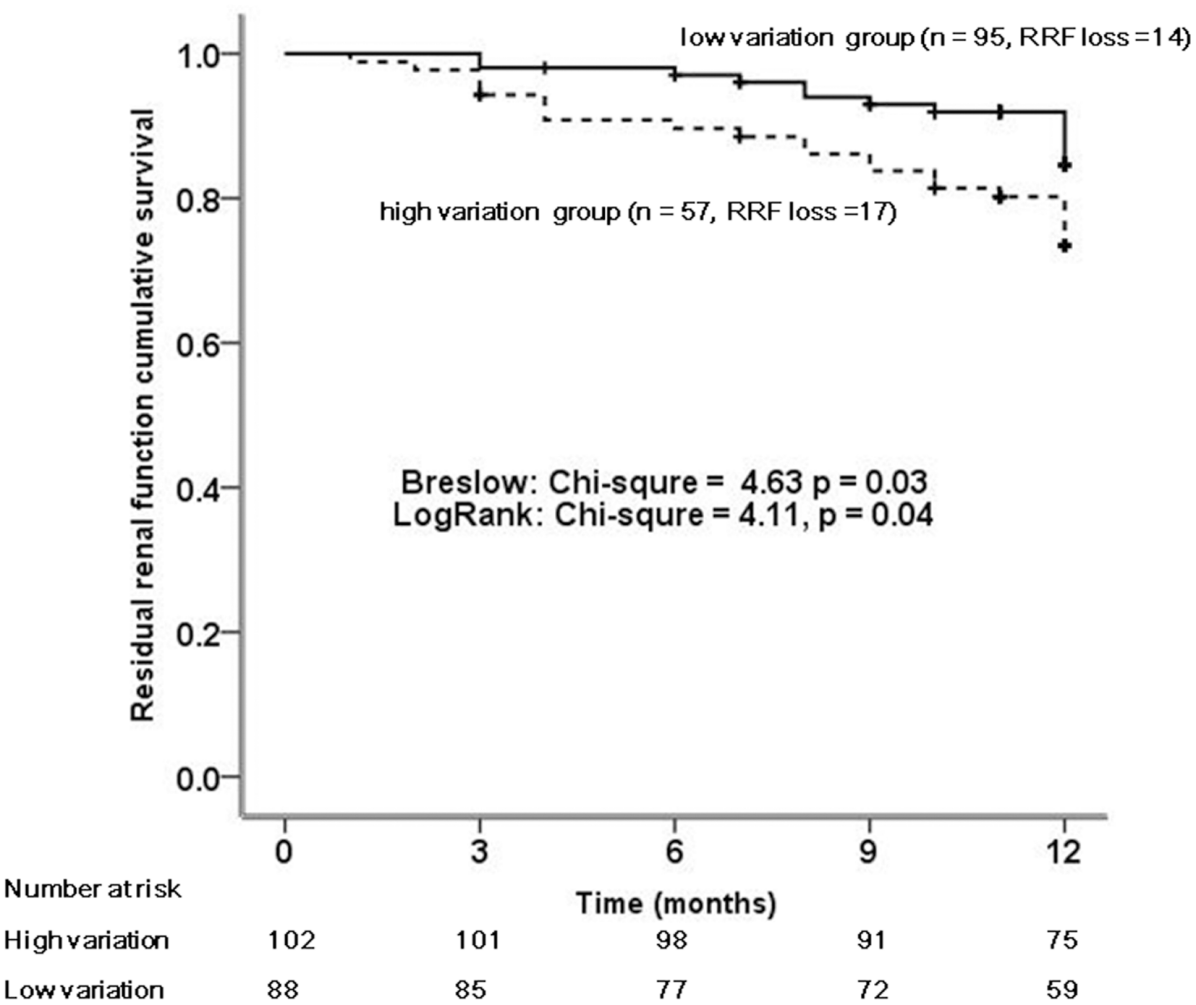
Kaplan-Meier survival curves for the residual renal function survival (event defined as anuria) compared between the high fluid variation group and the low fluid variation group.

The fluid variation was the highest in the IO group (Table 1). The decline rates of mGFR and urine volume in the high fluid variation group were significantly faster than those in the low fluid variation group (median mGFR decline rate: -0.2 vs -0.1 ml/min/1.73m^2^/month, *p* = 0.03; median of urine volume decline rate: -31.2 vs -17.5 ml/24h/month, *p* = 0.004) (data not shown). In the patients with high and low fluid variation, Kaplan-Meier curve showed that the patients in high variation group had significantly poorer RRF survival than those in low variation group (Log-rank chi-square = 4.1, *p* = 0.04) (Fig 3). Multivariable Cox proportional hazard model showed the fluid variation was not independently associated with RRF survival (Table 3).

**Table 3:** Cox regression analysis of risk factors associated with anuria.

variables	univariate		multivariate	
	HR (95%CI)	*p*	HR (95%CI)	*p*
Mean ECW/TBW (↑0.01)	1.86(1.39–2.49)	<0.001	2.05(1.38–3.06)	<0.001
SD of ECW/TBW (↑0.001)	1.10(1.01–1.20)	0.03	1.01(0.91–1.12)	0.8
Male	0.70(0.36–1.35)	0.2	-	-
Age (↑1 yr)	0.98(0.96–1.00)	0.1	0.95(0.92–0.97)	<0.001
Vintage(↑1 month)	0.99(0.97–1.01)	0.5	-	-
Diabetes	1.03(0.51–2.11)	0.9	-	-
Charlson co-morbidity score (↑1)	1.04(0.86–1.24)	0.6	-	-
SBP (↑10mmHg)	1.02(1.00–1.03)	0.003	1.02(1.00–1.03)	0.02
Baseline urine volume(↑100ml/d)	0.47(0.37–0.60)	<0.001	0.77(0.67–0.88)	<0.001
Serum albumin (↑1g/L)	0.96(0.89–1.03)	0.2	-	-
PD dosage (↑1L)	2.29(1.43–3.67)	0.001	2.78(1.16–6.66)	0.02
Solution glucose concentration (↑0.1%)	1.12(1.02–1.23)	0.01	0.96(0.26–3.59)	0.9
Ultrafiltration (↑100ml)	1.12(1.05–1.19)	<0.001	1.12(1.03–1.22)	0.008
Antihypertensive drug category (<3 vs ≧3)	0.56(0.29,1.09)	0.1	1.20(0.56,2.55)	0.6
ACEI/ ARB use	0.73(0.37–1.42)	0.3	-	-
Diuretics use	1.27(0.45–3.60)	0.6	-	-

Note: Factors with *p* value<0.1 in univariate analysis and gender were selected for the multivariate COX proportional hazard model.

Abbreviation: ECW: extracellular water, TBW: total body water, SBP: systolic blood pressure, SD: standard deviation, ACEI:angiotensin-converting enzyme inhibitor, ARB: angiotensin receptor blocker.

### Risk Factors on RRF Decline

Higher ECW/TBW, age, higher systolic blood pressure, lower urine volume at baseline, more PD dosage and more ultrafiltration were independently associated with an increased risk of RRF loss. Every 0.01-increment of mean ECW/TBW was significantly associated with a 2.05-fold increasing of RRF loss (HR = 2.05, 95% CI 1.38–3.06), after adjusted for selected variables in univariable COX regression analysis. Regardless of the adjustment method used through model 1 to 3, fluid status of the IO and PO group was independently associated with the loss of RRF compared to the NH fluid status. In model 3, which was a maximally adjusted model including gender, age, vintage, diabetes, systolic blood pressure, baseline urine volume, serum albumin, ACEI/ARB, and variation of fluid, the adjusted HR for IO and PO group were 5.62 (95% CI 1.62–19.39, *p* = 0.006) and 9.90 (95% CI 3.07–31.89, *p* < 0.001), respectively (Table 4).

**Table 4:** Multivariable Cox regression results showing the effect of PO and IO on anuria.

	Model 1		Model 2		Model 3	
	HR(95%CI)	*p*	HR(95%CI)	*p*	HR(95%CI)	*p*
NH	ref		ref		ref	
IO	4.39(1.56–12.36)	0.005	6.44(2.05–20.24)	0.001	5.62(1.62–19.39)	0.006
PO	9.20(3.15–26.82)	<0.001	10.59(3.35–33.46)	<0.001	9.90(3.07–31.89)	<0.001

Note: Model 1: adjusted for gender, age, vintage, diabetes; Model 2: adjusted for model 1 and systolic blood pressure, baseline urine volume, serum albumin, ACEI/ARB, ultrafiltration; Model 3: adjusted for model 2 and variation of fluid status (as a continuous variable).

Abbreviations: HR: hazard ratio, CI: confidence interval, PO: persistent overhydration, IO: intermittent overhydration, NH: normal hydration group, ACEI:angiotensin-converting enzyme inhibitor, ARB: angiotensin receptor blocker.

## Discussion

The results of the present study indicated that patients with fluid overload, either persistent or intermittent fluid overload, were associated with poor outcome of RRF. The adjusted HR for RRF loss with per 0.01-increase in mean ECW/TBW was 2.05 (95% CI 1.38–3.06, *p*<0.001) in PD patients, being independent of several potential confounders.

Whether hypervolemia or euvolemia is benefit to maintaining RRF has been a matter of debate. In the present study, we found that stable normal volume status assessed by over time BIA measurements was associated with better RRF outcome, while expanded volume was independently associated with faster RRF decline. Indeed, our previous cross-sectional study showed volume overload was inversely correlated with residual urine volume [8]. Van Biesen et al found that RRF outcome improved with higher volume removal, and decreased if the patients had hypertension, as a sign of volume overload [26]. Fan S et al also indicated that volume overload assessed by ECW/TBW was associated with loss of RRF in a cross-sectional study [27]. However, in previous studies, only the baseline ECW/TBW ratio was employed to determine fluid status [28,29,30], while in the present study, ECW/TBW ratio was tested at the baseline and different time-points during follow-up period, which may be more accurate to evaluate the fluid status during a longitudinal period. Recently, McCafferty et al [16] reported that fluid status measured by BIA at baseline and the end of follow-up was not associated with preservation of RRF in 237 PD patients. However, their retrospective longitudinal study at least proved that extracellular volume expansion was not favorable for protecting RRF. The reasons of the discrepancy between our study and previous reports may due to different research design. First of all, fluid status represented by ECW/TBW ratio was measured at baseline and continuously monitored every 3 months for one year in our study. Secondly, the primary endpoints of this study included both decline rate of RRF and onset of anuria. Furthermore, in this study, a threshold of ECW/TBW ≧ 0.4 was set to define fluid overload, which was shown to be closely related to RRF decline.

It is not surprising that patients with fluid overload were associated with poor baseline general condition (Table 1). They had older age, higher percentage of diabetes, faster peritoneal transport, higher blood pressure, more malnutrition and co-morbidities, which was consisted with reports by other investigators [22,31,32]. Naturally, the underlying conditions of health should certainly be considered as confounders affecting RRF outcome. Therefore, we built a multivariate model including diabetes, blood pressure, and nutrition status in this study. As shown in Table 3, our results indicated that fluid overload (increasing ECW/TBW) was independently associated with the loss of RRF after adjusted for those confounders. Definitely, many confounders couldn’t be controlled in a retrospective study. A prospective randomized clinical trial is warranted to testify this hypothesis.

In clinical practice, variation of fluid is frequently observed in dialysis patients with either expanded or normal volume. The time-varying changes of fluid status may be a deteriorating factor for the loss of RRF, which was strongly evidenced by the fast decline of RRF due to dramatically change in extracellular water in the process of hemodialysis [7,33]. In this study, the variation of fluid status was found to be the most significant in the IO patients, and its inverse association with the loss of RRF was indicated through Kaplan-Meier analysis and univariable Cox regression analysis. Although this significance disappeared in multivariate analysis (Table 3), it still hinted that fluid variation may be an important factor for RRF decline and the volume should be carefully controlled for an optimal balance. Of note, normal fluid status was benefit to preserve RRF, neither hypervolemia nor hypovolemia. Volume depletion, by itself, might accelerate the decline of RRF [26]. Actually, overuse of hypertonic solutions may lead to intravascular volume depletion and hypotension, which are known to cause RRF loss [12]. In this study, hypotension events were tried to be prevented carefully in the process of management on fluid overload. The result that faster declining of RRF was related to higher fluid status and higher fluid variation highlighted that more emphasis should be placed to maintaining normal and stable fluid status in PD patients.

“Volume first” should be a primary goal of dialysis care [34]. Actually, the results of our investigation suggested volume overload not only increase cardiovascular mortality [35,36], but also play an inverse role in the preservation of RRF. Therefore, keeping PD patients in a slight volume expansion may be an unfavorable strategy for protecting RRF. On the contrary, we should be more cautious about the high prevalence of fluid overload, balancing (sodium and water) intake and removal, and avoiding violent fluctuation of blood pressure and iatrogenic fluid variation.

There are some limitations in this study. Given the nature of a retrospective study, first, the causality of fluid status on RRF loss cannot be clarified affirmatively. Second, the imbalance among study groups was inevitable although the confounders were adjusted by the statistical analysis. Third, lacking precisely measurement of sodium intake and excretion made it fail to assess the sodium restriction accurately. Therefore, a prospective interventional study is warranted to answer whether volume expansion would lead to RRF decline or not.

## Conclusions

In conclusion, we found that fluid overload was closely associated with the deterioration of RRF. The association remained robust despite adjustment for the possible confounders. Further studies will be necessary to determine whether reducing volume overload can improve the survival of RRF in PD patients.

## Supporting Information

S1 File STROBE checklist. (PDF)
